# Treatment delay and associated factors among adults with drug resistant tuberculosis at treatment initiating centers in the Amhara regional state, Ethiopia

**DOI:** 10.1186/s12879-019-4112-2

**Published:** 2019-05-31

**Authors:** Kenaw Tegegne Tefera, Nebiyu Mesfin, Mebratu Mitiku Reta, Malede Mequanent Sisay, Koku Sisay Tamirat, Temesgen Yihunie Akalu

**Affiliations:** 1 University of Gondar Comprehensive Specialized Hospital, Gondar, Ethiopia; 2 Department of Internal Medicine, School of Medicine, College of Medicine and Health Sciences, University of Gondar, Gondar, Ethiopia; 3 Department of Epidemiology and Biostatistics, Institute of Public Health, College of Medicine and Health Sciences, University of Gondar, Gondar, Ethiopia

**Keywords:** Drug resistant tuberculosis, Treatment delay, Risk factors, Amhara region

## Abstract

**Background:**

A delayed initiation of tuberculosis treatment results in high morbidity, mortality, and increased person-to-person transmissions. The aim of this study was to assess treatment delay and its associated factors among adult drug resistant tuberculosis patients in the Amhara Regional State, Ethiopia.

**Methods:**

An institution based cross-sectional study was conducted on all adult drug resistant tuberculosis patients who initiated treatment from September 2010 to December 2017. Data were collected from patient charts, registration books, and computer databases using abstraction sheets. The data were entered using Epi-info version 7 and exported to SPSS version 20 for analysis. Summary statistics, like means, medians, and proportions were used to present it. Binary logistic regression was fitted; Adjusted Odds Ratio (AOR) with a 95% Confidence Interval (CI) was also computed. Variables with *p*-value < 0.05 in the multi-variable logistic regression model was declared as significantly associated with treatment delay.

**Results:**

The median time to commence treatment after drug resistant tuberculosis diagnosis was 8 (IQR: 3–37) days. Being diagnosed by Line probe assay [AOR = 5.59; 95% CI: 3.48–8.98], Culture [AOR = 5.15; 95% CI: 2.53–10.47], and history of injectable anti-TB drugs [AOR = 2.12; 95% CI: 1.41–3.19] were associated with treatment delays.

**Conclusion:**

Treatment delay was long, especially among patients diagnosed by Culture or LPA and those who had a prior history of injectable anti-TB drugs. That suggested that the need for universal accesses to rapid molecular diagnostic tests, such as Gene Xpert and the PMDT team were needed to promptly decide to minimize unnecessary delays.

## Background

Drug resistant tuberculosis (DR-TB) has been one of the leading public health problems globally [1]. There were an estimated 580,000 new cases and 230,000 deaths from multi-drug resistant tuberculosis (MDR-TB) according to the 2018 global TB report [2]. Thirty high drug resistant tuberculosis burden countries shared more than 85% of the global DR-TB prevalence [1]. About 20% of previously treated and 4.1% of the new patients had drug resistant tuberculosis [1, 3, 4]. Ethiopia is one of the highest drug resistant TB burden countries with an estimated 2100 MDR-TB cases annually [5]. Drug resistant TB treatment is long, extending from 18 to 24 months on toxic drugs. Successful treatment outcomes of DR-TB patients remain around 50% [4, 6].

Effective TB prevention and control was achieved through early case detection and prompt treatment initiation [7]. Different evidences show that timely initiations of drug resistant tuberculosis treatments are of paramount importance for increasing favorable outcomes and halting person to person transmissions [8]. In Ethiopia however TB case detection rate which was characterized by delayed treatment commencement after diagnosis was 64% [9–11]. Treatment delay has been one of the major factors imposing a challenge to TB prevention and control [12]. Delayed treatment initiation leads to increased morbidity, mortality, and the progression of the disease to severe and complicated forms [13–15]. Besides, patients with cavitary lesions and high bacilli loads are associated with an increased primary form of drug resistant TB and rapid person-person transmissions [16].

Treatment delays which were studied in both high and low income countries significantly varied from 9 days in China [8] to 19 days in India [17], and from 10 days in South Africa [18] to 18 days in Zimbabwe [19]. Patient related factors such as health seeking behavior and distance from health facilities from residences [18] as well as the history of anti-TB treatment [8, 15] and diagnostic modalities [6, 20, 21] were significantly associated with delayed treatment initiations. In addition, health facility factors were contributing to treatment delays due to inadequate treatment initiating centers (TICs) with limited beds [22].

The World Health Organization (WHO) has conditionally recommended the ambulatory model of care for a timely initiation of drug resistant TB treatment [23]. The Ethiopia Federal Ministry of Health has been expanding ambulatory DR-TB treatment centers. Though some studies have been conducted in other high burden countries, evidence of treatment delays of DR-TB patients is still scarce.

Therefore, this study aimed to determine treatment delays and its associated factors in the Amhara Regional State. The study could provide insight about treatment delays to clinicians and other stakeholders.

## Methods

### Study design and setting

An institution-based cross-sectional study was conducted at the drug resistant TB treatment initiation centers in the Amhara Regional State from September 2010 to December 2017. In the region, four of the nine hospitals, namely University of Gondar Comprehensive Specialized, Borumeda, Woldiya, and Debre-Markos which provided the service to more than 90% of the DR-TB patients and kept data for a long time were selected. While the remaining five hospitals were opened later which lacks samples and organized data that makes randomization difficult in the selection of hospitals. Besides, the four included hospitals were found in the main cities of the region and can be considered as representative of the region. Furthermore, the recent five centers were located in the districts and their main purpose was to support the four main TICs as outpatient follow up areas and enhance accessibility of services at district levels.

Drug sensitivity test for MDR-TB patients was done using Gene Xpert, Line Probe Assay (LPA), and culture. All of the selected hospitals had Gene Xpert machines and tests were done onsite so that there was no need of transportation of specimens. In contrast, all hospitals which had no culture and LPA tests and samples had to transport specimen to the Regional Research and Laboratory Institute. So, they used the postal system for transportation of specimens. Similarly, they received feedback via the post office. These may have their own contributions to treatment delays. But, University of Gondar comprehensive specialized and Borumeda hospitals introduced culture and LPA recently.

The Programmatic Management Committee of Drug Resistant Tuberculosis (PMDT) is a multi-disciplinary team composed of physicians, nurses, and TB program officers who discussed and prepared patient treatment plans. The team meets regularly when new patient are enrolled for DR-TB treatment, at the end of intensive phase, and at the completion of treatments for final evaluation.

### Population and sample

All bacteriologically confirmed adult DR-TB patients who initiated treatment during the study were included. Parameters which are important input for sample size determination were not available. Hence, we assumed proportion (P) for treatment delay 50, 95% level of confidence, 5% margin of error, and 5% non-response rate. These yielded a final sample size of 403. However, in the four selected hospitals, we had a total of 546 participants 16 of whom were excluded due to missing of the outcome variable. Finally, 530 respondents who met the inclusion were considered in the final analysis.

### Data collection and variables of the study

Data were collected using an extraction checklist prepared in English. Six data collectors and three supervisors (nurses and health officers) were recruited. Two days training was given on the objectives of the work and how to review documents before the process. Prior to data collection, records (both baseline and follow up) were reviewed and identified by their medical registration/card numbers. Trained collectors reviewed and extracted data from patient medical charts and computer database.

In this study, treatment delay measured in number of days was the dependent variable, whereas socio-demographic characteristics, like sex, age, residence, educational status, ethnicity, marital status, occupation and religion were collected. Behavioral factors that included smoking, chat-chewing, and alcohol drinking were gathered**.** We also collected clinical characteristics, like HIV co-infection, TB treatment history, chief complaint, history of injectable anti-TB drug, co-morbidity, site of DR-TB, base line sputum culture and smear result, baseline BMI, functional status and diagnostic modalities like Gene Xpert, LPA, and culture.

Treatment delay expressed as the median number of days from date of DR-TB laboratory confirmation to commencement of treatment. Since there was no standardized definition for treatment delay in Ethiopia, we used the median number of days as a cut off point for categorizing as delayed and not delayed. Patients who initiate DR-TB treatment after 8 days was classified as treatment delay.

Bacteriologically confirmed DR-TB was defined as patients who were resistant to at least Rifampicin and other first line anti-Tb drugs, this means if a patient was resistant to at least Rifampicin alone or Rifampicin and a combination of any of the first line anti-TB drugs.

New refers to those patients who were never treated for TB or took anti-TB drugs for < 1 month.

Previously treated: Patients who treated for TB for one or more months.

Multidrug resistance (MDR-TB): TB resistant to at least both Isoniazid and Rifampicin.

Rifampicin Resistant TB: TB resistant to Rifampicin detected using phenotypic or genotypic methods, with or without resistance to other anti-TB drugs.

Clinic-based ambulatory model of care is a care designed to deliver the treatment course on outpatient basis as long as the patient is fit to be followed as ambulatory.

Treatment initiating centers (TICs) are health facilities selected by the TB program to provide patient care and treatment services right from the time of DR-TB diagnosis and throughout the course of treatment with SLDs.

Body mass index (BMI) was defined as severely low when BMI < 16, low 16–18.49 kg/m^2^, normal 18.5–24.99 kg/m^2^, overweight 25–29.99 kg/m^2^, and obese > 30 kg/m^2^.

### Data processing and management

Data were checked for completeness and entered using Epi Info version 7 and exported to SPSS version 20 for analysis. Categorical variables were summarized by counts and percentages, and the differences between groups were compared using chi-square (*x*^2^). The binary logistic regression model was fitted by considering treatment delay as an outcome of interest. A bi-variable logistic regression model was first fitted, and variables that were significant at p < 0.2 in the bi-variable analysis were entered into the multi-variable logistic regression model. Crude and adjusted odds ratios with 95% confidence interval (CI) were used to determine the strength of association between the dependent and independent variables. Variables with *p*-value < 0.05 in the multi-variable model were considered as statistically significant predictors of drug resistant tuberculosis treatment delay.

## Result

### Socio-demographic characteristics

A total of 546 confirmed adult DR-TB patients were registered and started on DR-TB treatment during the study. Of these, 530 (97%) DR-TB patients who had complete records were included in the analysis. More than half (57%) of the patients initiated treatment at the University of Gondar comprehensive specialized hospital. The median age of patients was 29 (IQR: 24–40) years. The majority of the participants were Orthodox Christian (83.4%); more than half (53.8%) were from urban areas, and 56.2% were male; about half (50.6%) were married. Nearly one third (29.8%) of the patients were unable to read and write, and 28.7% were farmers. Eighty three (15.7%) and 13.4% of the patients had history of alcohol use and smoking, respectively (Table 1).

**Table 1:** Baseline Socio-demographic characteristics of adult DR-TB patients in Amhara regional State treatment initiating centers from September 2010 to December 2017 (*n* = 530)

Variables		Frequency (%)
Hospitals (TICs)	Gondar University Hospital	302 (57.0%)
	Borumeda Hospital	136 (25.7%)
	Debre-Markos Hospital	41 (7.7%)
	Woldiya hospital	51 (9.6%)
Sex	Male	298 (56.2%)
	Female	232 (43.8%)
Age (years)	18–24	152 (28.7%)
	25–29	114 (21.5%)
	30–40	160 (30.2%)
	= > 41	104 (19.6%)
Residence	Urban	285 (53.8%)
	Rural	245 (46.2%)
Ethnicity	Amhara	77 (90%)
	Tigre	25 (4.7%)
	Other^a^	28 (5.3%)
Religion	Orthodox	442 (83.4%)
	Muslim	88 (16.6%)
Educational status	Cannot read and write	158 (29.8)
	Primary	215 (40.6)
	Secondary	86 (16.2)
	Tertiary	71 (13.4)
marital status	Married	268 (50.6%)
	Single	172 (32.5%)
	Others^b^	90 (8.9%)
Occupation	Employed^c^	139 (26.2%)
	Unemployed^d^	239 (45.1%)
	Farmer	152 (28.7%)

Others^a^ Oromo, Kimant, Afar

Others^b^ Divorced, Widowed, separated

Employed^c^ Government, Non-Government and private

Unemployed^d^ daily laborer, house wife, and student

### Clinical characteristics

The majority, (93.0%) of the patients had pulmonary TB; 77.2% had positive sputum smear results at baseline. Regarding laboratory confirmation, 58.3% were confirmed by Gene-expert, followed by LPA and culture. Most of the patients (83.4%) had previous TB treatment history, with a mean of 2.5 prior treatment. Of the previously treated patients, nearly 42.3% had history of injectable anti-TB drugs. Nearly one-fourth (27%) of the patients were HIV co-infected. Nearly three-fourth (73%) had low BMI, and about 47.7% were ambulatory by functional status at the initiation of treatment (Table 2).

**Table 2:** Baseline Clinical characteristics of adult DR-TB patients in Amhara regional State treatment initiating centers from September 2010 to December 2017 (n = 530)

Variable		Frequency (%)
Base line BMI	Low	387 (73.0%)
	Normal	142 (26.8%)
Chief complain	Cough	489 (92.3%)
	Others^a^	41 (7.7%)
History of TB treatment	New	88 (16.6%)
	Previously treated	442 (83.4)
History of injectable anti-TB drug	Yes	224 (42.3%)
	No	306 (57.7%)
Site of DR-TB	Pulmonary	493 (93.0%)
	Extra pulmonary	37 (7.0%)
base line sputum smear result	Positive	409 (77.2%)
	Negative	121 (22.8%)
DR-TB diagnosed by	Gene Expert	309 (58.3%)
	LPA	165 (31.1%)
	Culture	56 (10.6%)
Base line functional status	Working	194 (36.6%)
	Ambulatory	253 (47.7%)
	Bedridden	83 (15.7%)
HIV status	Positive	143 (27.0%)
	Negative	387 (73.0%)

Treatment delay^*^---- in this study was considered as those DR-TB patients commenced treatment after eight days of diagnosis

Others^**^------Chest pain, Weight loss, LN swelling, SOB …

*BMI* Body mass index, *LPA* Line probe Assay, *TB* Tuberculosis

**Table 3:** Bi-variable and Multivariable Binary logistic regression analysis for factors associated with treatment delay among adult DR-TB patients in Amhara regional State treatment initiating centers from September 2010 to December 2017 (*n* = 530)

Variable		Treatment delay		OR (95% CI)	
		Yes	No	COR	AOR
TIC	GUH	165	137	3.52(1.80–6.88)	1.41(0.64–3.11)
	Borumeda hospital	59	77	2.24(1.09–4.58)	1.33(0.56–3.17)
	Debre-Markos hospital	19	22	2.52(1.05–6.08)	1.45(0.55–3.79)
	Woldiya hospital	13	38	1	1
Occupation	Employed	64	75	1	1
	Unemployed	109	130	0.98 (0.65–1.49)	0.84(0.51–1.38)
	Farmer	83	69	1.41(0.89–2.24)	1.14(0.66–1.97)
Educational status	Cannot read and write	77	81	0.78 (0.45–1.37)	0.89(0.46–1.73)
	Primary	96	119	0.66 (0.39–1.14)	0.55 (0.29–1.04)
	Secondary	44	42	0.86 (0.46–1.61)	0.81(0.39–1.69)
	Tertiary and above	39	32	1	1
Base line functional status	Working	77	117	1	1
	Ambulatory	143	110	1.98(1.35–2.89)	1.03(0.64–1.68)
	Bedridden	36	47	1.16(0.69–1.96)	0.89(0.46–1.72)
Previous TB Rx history	New	24	64	1	1
	One times and above	232	210	2.95(1.78–4.88)	1.23(0.69–2.19)
HIV status	Positive	55	88	0.58(0.39–0.86)	0.69(0.44–1.11)
	Negative	201	186	1	1
Type of diagnostic test	Expert	92	217	1	1
	LPA	122	43	6.69(4.38–10.23)	5.59(3.48–8.98)*
	Culture	42	14	7.08(3.69–13.58)	5.15(2.53–10.47)*
Chief complain	Cough	241	248	1	1
	Others	15	26	0.59(0.31–1.15)	0.65(0.29–1.45)
History of injectable anti-TB drug	Yes	139	85	2.64(1.85–3.77)	2.12(1.41–3.19)*
	No	117	189	1	1
Base line BMI	Low	177	210	0.69(0.47–1.02)	0.75(0.48–1.17)
	Normal	78	64	1	1

*OR* odds ratio, *CI* confidence interval, *LPA* Line Probe Assay

Note: * = shows statistically significant at *p*<0.05

### Treatment delay of drug resistant tuberculosis patients

**Fig. 1:**
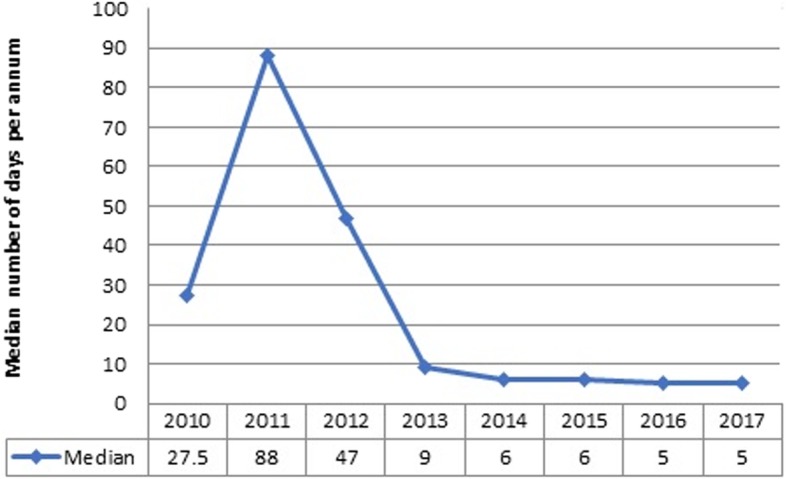
Trend of treatment delay among adult DR-TB patients in Amhara region treatment initiating centers from September 2010-Decmber 2017

The median time from diagnosis to initiation into anti-DR-TB treatment was 8 days (IQR: 3–37 days). Regarding treatment initiating centers, the University of Gondar had a median treatment initiation of 11 (IQR: 3–52) days, Borumeda of 6 (IQR: 1–20) days, Debre-Markos of 8 (IQR: 4–17.5) days, and Woldiya of 6 (IQR: 2–10) days. About 68 (12.8%) of the patients were initiated within one day after diagnosis, and nearly one third (27.4%) started treatment after 30 days and above. Delayed DR-TB treatment was reported for 48.3% [95% CI, 44.0–52.8%] of the patients. The median time to initiate DR-TB treatment decreased over time (Fig. 1).

### Factors associated with treatment delays

Findings from the bi-variable logistic regression analysis showed that treatment initiating centers, occupation, educational status, chief complaints, functional status, TB treatment history, diagnostic modalities, history of treatment with injectable anti-TB drugs, HIV co- infection, and base-line BMI were associated with treatment delay. In the multi-variable logistic regression analysis however only history of treatment with injectable anti-TB drugs and types of diagnostic modality remained significantly associated with the delays (Table 3).

The odds of treatment delay among patient who had history of treatment with injectable anti-TB drugs were two times higher [AOR = 2.12; 95% CI: 1.41–3.19, *P* < 0.0001] compared with patients with no such history. The odds of treatment delays among patients diagnosed by culture were 5 times higher [AOR = 5.15; 95% CI: 2.53–10.47, P < 0.0001] and by LPA 5.6 times higher [AOR = 5.59; 95% CI: 3.48–8.98, P < 0.0001] compared with patients diagnosed by Gene Expert.

## Discussion

This study showed the median time to initiate drug resistant tuberculosis treatment after a confirmed diagnosis was 8 days. Previous history of injectable drug use and diagnostic modalities were independent predictors of delays. The median time to initiate DR-TB treatment in this study was shorter than those of studies conducted in Bangladesh (median of 10 days) [22], Gauteng, South Africa (median of 10 days) [24], and Harare, Zimbabwe (median of 18 days) [19]. This might be due to differences in sample sizes, drug resistant tuberculosis diagnostic modalities, health care systems, and distance from reference laboratory which affects result turnaround time.

Treatment initiations among DR-TB patients mainly depended on baseline laboratory investigations [6], PMDT panel team decisions, the availability of drugs, and the DST result of patients [25]. Accordingly, Fig. 1 shows that the median time to initiate drug resistant tuberculosis treatment significantly decreased from a median of 27.5 and 88 days in 2010 and 2011 to 5 days in 2017. The possible explanation might be the introduction of new diagnostic methods, like Gene Xpert, which decreased laboratory result turnaround time from national reference laboratories. Gene Xpert provides worthy advantages for both mycobacterium case detection and Rifampicin resistance pattern identification within a short period of time [26]. Furthermore, the DR-TB treatment policy revision from hospital based to ambulatory model of care in health facilities helps in the reduction of waiting time for admission and commencement of treatment. Though improvements had been made to reduce treatment delay overtime, still greater efforts are needed to reduce delay to below 8 days.

Patients who had history of injectable anti-TB drugs from previous tuberculosis treatment courses were two times more likely to have treatment delays than patients had no prior history of injectable anti-TB drug use. This study was supported by a previous report from India [14]. The majority of the patients in this study had tuberculosis treatment experience on category-II streptomycin containing regimen resulting from poor implementations of DST among presumptive DR-TB patients. Patients experiencing injectable anti-TB drug from previous treatment courses had complaints of injection site pain and abscess. In addition, such patients frequently developed serious side effects, especially hearing impairment and renal function deterioration [27, 28]. Hence, PMDT team follow up renal function tests and hearing assessments before the initiation of treatment, frequently. Second line anti-TB drugs are highly toxic with severe and life-threatening adverse effects which result in reducing dosing intervals or suspending temporally the second line injectable anti-TB drug until patients recover. This may lead to late initiation of treatment compared to patients who had not previous injectable anti-TB drugs. This finding suggests that further improvement be made in the treatment initiation through adequate counselling and close follow ups early after diagnosis before the patient status declines as a result of fatigue from previous injectable anti-TB drugs. Currently the WHO approved short regimen reduced treatment duration to 9–12 months and the incidence of adverse drug effects [29]. Unfortunately, no patient was on Bedaquiline and Delanamide containing short regimen during our data collection period.

Drug resistant tuberculosis diagnostic modalities were significantly associated with treatment delay among DR-TB patients. Thus, patients diagnosed by conventional culture and Line probe assay (LPA) experienced treatment delays. A similar finding was observed in studies done in Europe [15, 20, 30], Asia [7, 21, 31], and Africa [19, 32–34]. This may be so because participants were diagnosed by Gene Xpert done directly from sputum with no need for prior smear examination and facilitated the simultaneous detection of MTB and Rifampicin resistance. It provides results within two hours instead of several days and weeks unlike LPA and culture, respectively. LPA may also take several weeks in smear negative patients since culture is still needed prior to LPA examination such patients [28]. In addition, Gene Xpert takes less time to send results back to clinicians because the test is done onsite from TICs by themselves while culture and LPA are performed in reference laboratories located some distance from the TICs [21]. Some evidence from Sub-Saharan Africa also showed that the use of the Gene Xpert diagnostic modality resulted in the rapid initiation of tuberculosis treatment within same day as the diagnosis along with timely information about the presence or absence of Rifampicin-resistance [16, 35]. Therefore, this suggests that the need to scale up simple and rapid molecular diagnostic tests, like Gene Xpert that can be used at the lowest health care facilities. The finding supports the strategy to scale up this test at service delivery points to achieve early initiation of treatments.

In contrast to our finding, previous studies indicated that time from diagnosis to treatment initiation did not differ significantly among the three diagnostic modalities [12, 36, 37]. Several other studies showed that different socio-demographic factors [4, 22] and clinical characteristics [6, 8, 15] were associated with treatment delays. In however our study socio-demographic characteristics of patients (sex, age, education, occupation, residence, /rural or urban), and clinical characteristics, like baseline BMI, HIV/AIDS, and extra pulmonary TB or pulmonary TB were not associated with delays.

## Limitations

Firstly, since we have used secondary data, we could not address all potential variables like average monthly income, distance from TICs, and time took to reach the centers. Secondly, the fact that no WHO of national cut off point available to determine the impact of delays DR-TB patients made the comparison of main findings of the work difficult. Thirdly, differences in the diagnosis modalities relating to conforming treatment history by clinicians and biases due to missing and outcome variables might have resulted in an overall misclassification.

## Conclusion

Treatment delay was long, especially among patients diagnosed by Culture or LPA and those who had prior history of injectable anti-TB drug. This suggests the need for universal accesses of rapid molecular diagnostic tests, such as Gene Xpert, and the PMDT teams need to decide promptly to minimize unnecessary delays.

## Data Availability

Data is available from the corresponding author upon request.

## Abbreviations

AOR: Adjusted Odds Ratio
BMI: Body Mass Index
CI: Confidence Interval
DR-TB: Drug resistance Tuberculosis
DST: Drug Sensitivity Test
GUH: Gondar University Hospital
HIV: Human Immune Deficiency Virus
IQR: Inter Quartile Range
LPA: Line Probe Assay
MDR-TB: Multi-Drug Resistance Tuberculosis
MTB/RIF: Mycobacterium Tuberculosis/Rifampicin
PMDT: Programmatic Management of Drug Resistance Tuberculosis
SLD: Second line ant-TB Drugs
SPSS: Statistical Package for Social Science
TB: Tuberculosis
TIC: Treatment Initiating Center
WHO: World Health Organization
